# RC48-ADC treatment for patients with HER2-expressing locally advanced or metastatic solid tumors: a real-world study

**DOI:** 10.1186/s12885-023-11593-9

**Published:** 2023-11-09

**Authors:** Ping Wang, Lei Xia

**Affiliations:** 1 Department of Cancer Center, The Second Affiliated Hospital of Chongqing Medical University, Chongqing, 401336 China; 2 Tianjin Key Laboratory of Radiation Medicine and Molecular Nuclear Medicine, Institute of Radiation Medicine, Chinese Academy of Medical Sciences and Peking Union Medical College, Tianjin, 300110 China; 3 Department of Radiation Oncology, Key Laboratory of Cancer Prevention and Therapy, Tianjin Medical University Cancer Institute and Hospital, National Clinical Research Center for Cancer, Tianjin’s Clinical Research Center for Cancer, Tianjin, 300060 China

**Keywords:** RC48-ADC, HER2, Solid tumors, Disease control rate, Progression-free survival, Safety

## Abstract

**Background:**

RC48-antibody-drug conjugates (ADC) link humanized anti-HER2 immunoglobulin with monomethyl auristatin E (MMAE). Clinical trials suggest promising antitumor activity in HER2-expressing solid tumors. This study probes RC48-ADC’s efficacy and safety in patients with HER2-expressing advanced or metastatic solid tumors.

**Method:**

Data was collected from 23 advanced cancer patients treated with RC48-ADC at our oncology center between July 2021 and December 2022. These patients exhibited at least 1 + expression of HER2 immunohistochemistry, had previously experienced at least one failed systemic chemotherapy, and were treated with RC48-ADC until the occurrence of intolerable adverse reactions or disease progression. The primary endpoint was the disease control rate (DCR), and secondary endpoints included progression-free survival (PFS), objective response rate (ORR), and safety.

**Results:**

23 of 25 screened patients received RC48 treatment. The ORR was 43.5% (95% CI, 23.2-63.7%) with a median PFS of 6.0 months (95% CI, 4.8–7.4). In the low-to-medium HER2 expression subgroup, ORR was 37.5%, median PFS 5.75 months. In the high HER2 expression subgroup, ORR was 57.1%, median PFS 7 months. For the cohort combining RC48 with PD-1 inhibitors, ORR was 53.8%, median PFS 8 months. In the concurrent local radiation therapy subgroup, ORR was 40.0%, median PFS 6.0 months. Treatment-related adverse events (TRAEs) were anemia (60.8%), leukopenia (56.2%), raised transaminases (52.17%), and neutropenia (43.5%). Five patients (21.7%) experienced Grade 3 symptoms, including anemia (21.7%) and neutropenia (14.0%). No Grade 4 adverse reactions or deaths were reported.

**Conclusion:**

RC48-ADC shows promising efficacy and manageable safety in HER2-expressing advanced or metastatic solid tumor patients.

**Supplementary Information:**

The online version contains supplementary material available at 10.1186/s12885-023-11593-9.

## Background

Currently, a variety of targeted drugs for human epidermal growth factor receptor 2 (HER2)-expressing tumors have been developed, including antibody-drug conjugates (ADC), small molecule targeted drugs, and vaccines. Over the past several decades, targeted drugs for HER2 have been effective only in patients with HER2 amplification or overexpression and have not improved the prognosis of patients with low HER2 expression [1–3]. At present, Trastuzumab deruxtecan is the first approved antibody-drug conjugate for the treatment of low HER2-expressing breast cancer, and it has also shown good antitumor activity in low HER2-expressing solid tumors [4].

RC48 is a cutting-edge HER2-targeted antibody-drug conjugate, synthesized via the covalent bond of a novel recombinant human anti-HER2 monoclonal antibody and monomethyl auristatin E (MMAE), a microtubule inhibitor, through a cleavable linker [5]. Combining the precision of antibody targeting and the destructive power of small molecule drugs, RC48 accurately identifies and eradicates tumor cells [6]. After binding to the extracellular region of HER2 on the cell surface, the ADC complex is transported to lysosomes, where the linker undergoes enzymatic cleavage, releasing the microtubule inhibitor MMAE, which induces mitotic cell cycle arrest and cell apoptosis [7]. Concurrently, the liberated small molecule toxin MMAE eradicates neighboring HER2-negative or low-expression tumor cells, a process known as the “bystander effect,“ thereby overcoming the spatial heterogeneity of the tumor [7, 8]. As a result, RC-48 demonstrates similar efficacy in tumors with both low-expression and high-expression HER2 (HER2 IHC 2+ & FISH + or HER2 IHC 3+) [9].

Currently, Fam-trastuzumab deruxtecan-nxki (Vidextus) has been approved for the treatment of locally advanced or metastatic gastric cancer (including adenocarcinoma of the gastroesophageal junction) with HER2 overexpression, which has previously undergone at least 2 systemic therapies [10]. It has also been approved for indications in other types of cancer, such as urothelial carcinoma [11], and is currently under investigation in numerous clinical studies for diseases like breast cancer [12, 13] and lung cancer (NCT04311034). For instance, the RC48-C008 study is a clinical trial of Vidextus in the field of gastric cancer, incorporating 125 patients with advanced metastatic gastric cancer who have previously undergone ≥ 2 lines of treatment, including patients with HER2 IHC 2 + and 3 + expressions. The study reported an ORR of 24.8%, a DCR of 42.6%, a PFS of 4.1 months, and an overall survival (OS) of 7.9 months. In the RC48-C005 study, out of the 43 patients with second-line and beyond urothelial carcinoma treated with RC48-ADC, the ORR was 51.2%, DCR was 90.7%, PFS was 6.9 months, and OS was 13.9 months [10] .

We performed statistical analysis on the efficacy and safety of RC48-ADC treatment in 23 patients with HER2-expressing locally advanced or metastatic malignant solid tumors who were treated at our center.

## Materials and methods

### General information

We conducted a retrospective analysis of patients with locally advanced or metastatic HER2-expressing malignant solid tumors who failed first-line or multiple-line treatments at the Cancer Center of the Second Affiliated Hospital of Chongqing Medical University from July 2021 to December 2022. The inclusion criteria were as follows: (1) patients with locally advanced or metastatic solid tumors confirmed to express HER2 through immunohistochemical detection (IHC 1+, IHC 2+, IHC 3+); (2) aged above 18 years old; (3) progression of tumors after receiving first-line or multiple-line treatments; (4) at least one measurable lesion according to the Response Evaluation Criteria In Solid Tumors (RECIST1.1); and (5) Eastern Cooperative Oncology Group (ECOG) performance status score ≤ 2 points. The exclusion criteria were: (1) patients unwilling to accept follow-up; (2) patients with severe cardiovascular or cerebral diseases. According to the inclusion and exclusion criteria, 23 patients who received RC48-ADC treatment were selected.

### Treatment plan

All HER2-expressing patients underwent routine vital sign monitoring, blood tests, liver and kidney function tests, electrolyte tests, coagulation function tests, imaging examinations, etc., before treatment. Vidextamab (RC48-ADC), produced by Rongchang Biopharmaceuticals Ltd, was administered at a dosage of 2.5 mg/kg by intravenous infusion every two weeks. Safety assessments were performed each cycle, and efficacy evaluations every four cycles. RC48 treatment continued until disease progression, intolerable toxic side effects, voluntary withdrawal, or death occurred. After treatment completion, all participants were followed up every three months for survival until death or loss of follow-up.

### Efficacy assessment

Efficacy Evaluation Criteria: The Response Evaluation Criteria in Solid Tumors (RECIST 1.1 version) [14] developed by the National Institutes of Health (NIC) and the European Organisation for Research and Treatment of Cancer (EORIC) are utilized. These include Complete Response (CR): the disappearance of target lesions, pathological lymph node short diameter reduced to < 10 mm; Partial Response (PR): the sum of the diameters of target lesions reduced by 30% compared to baseline; Progression of disease (PD): at least a 20% increase in the sum of the diameters of all target lesions, emphasizing an absolute increase in the sum of diameters of more than 5 mm, or the appearance of new lesions; Stable Disease (SD): changes that fall between PR and PD. The Objective Response Rate (ORR) = (CR + PR)/N, Disease Control Rate (DCR) = (CR + PR + SD)/N, Progression-Free Survival (PFS): the time from enrollment to tumor progression or death.

### Adverse reaction Assessment

Adverse reactions are graded according to the World Health Organization (WHO) Common Terminology Criteria for Adverse Events (CTCAE), which ranges from grade 0 to 4 [15].

### Statistical analysis

Statistical analysis was performed using SPSS 26.0 software.

## Results

From July 2021 to December 2022, a total of 23 patients underwent RC48 treatment. The ages ranged from 36 to 80 years. There were 9 males and 14 females; 7 patients had an ECOG score of 0, 9 had a score of 1, and 7 had a score of 2; 11 patients had an IHC score of 1+, 5 had an IHC score of 2+, and 7 had an IHC score of 3+. Among these patients, 9 had breast cancer, 7 had gastric cancer, 3 had bladder cancer, 1 had colon cancer, 1 had cervical cancer, 1 had thyroid cancer, and 1 had duodenal cancer. All patients had visceral metastases, with lung metastases in 10 patients (43.5%) and liver metastases in 10 patients (43.5%). In this study, patients had received a median of three lines of prior systemic therapy, with a range spanning from one to five different regimens. Notably, 30.4% of these patients, all of whom were HER2-positive, had undergone previous HER2-targeted treatments, encompassing drugs such as Trastuzumab, Pertuzumab, and Lapatinib. It was universal among the cohort that they had a history of chemotherapy, predominantly involving agents from the taxane and platinum families. Furthermore, 39.1% of the participants, representing 9 out of 23 patients, had experience with PD-1 inhibitor therapy in the past. During the course of receiving RC48 treatment, ten patients (43.5%) received concurrent local radiation therapy, 13 received concurrent immunotherapy (PD-1 inhibitors), and 2 received concurrent anti-HER2 monoclonal antibody therapy (Table 1).

**Table 1:** The baseline characteristics of the study population

	All patients (N = 23)	HER-2 low (N = 16)	HER-2 positive (N = 7)
Age (years)			
Median	53	54.5	48
Min, Max	36, 80	39, 80	36,78
Gender			
Male	9 (39.1%)	8 (50%)	1 (14.3%)
Female	14 (60.7%)	8 (50%)	6 (85.7%)
ECOG status			
0	7 (30.4%)	5 (31.3%)	2 (28.6%)
1	9 (39.1%)	7 (43.7%)	2 (28.6%)
≥ 2	7 (30.4%)	4 (25.0%)	3 (42.8%)
Cancer type			
Breast	9 (39.1%)	3 (18.8%)	6 (85.7%)
Gastric	7 (30.4%)	7 (43.7%)	0
Colorectal	1 (4.3%)	1 (6.2%)	1 (14.3%)
Urothelial	3 (13.1%)	2 (12.5%)	0
other	3 (13.1%)	3 (18.8%)	0
Number of metastatic sites			
1–2	15 (63.2%)	12 (75.0%)	3 (42.8%)
≥ 3	8 (34.8%)	4 (25.0%)	4 (57.2%)
Metastasis sites			
Lung	10 (43.5%)	7 (43.7%)	3 (42.8%)
Liver	10 (43.5%)	6 (37.5%)	4 (57.2%)
Brian	5 (21.7%)	1 (6.2%)	4 (57.2%)
Bone	8 (34.8%)	6 (37.5%)	2 (28.6%)
HER-2 expression			
IHC 3 + or IHC 2 + FISH+	7 (30.4%)	0	7 (100%)
IHC 2 + FISH-	4 (17.4%)	4 (25.0%)	0
IHC 2 + FISH unknown	1 (4.3%)	1 (6.2%)	0
IHC 1+	11 (47.8%)	11 (68.8%)	0
Number of prior systemic therapies			
One prior line	8(34.7%)	7 (43.7%)	1 (14.3%)
Two prior line	3 (13.1%)	2 (12.5%)	1 (14.3%)
Three prior line	5 (21.7%)	4 (25.0%)	1 (14.3%)
Four prior line	4 (17.4%)	2 (12.5%)	2 (28.6%)
Five prior line	3 (13.1%)	1 (6.2%)	2 (28.6%)
Prior HER2-targeted therapy	7 (30.4%)	1 (6.2%)	6 (85.7%)
Combined radiotherapy	10 (43.5%)	6 (37.5%)	4 (57.2%)
Co-medication status			
No co-medication	6 (26.1%)	3 (18.8%)	3 (42.8%)
PD- 1 or PD-L1 inhibitors	13 (56.5%)	12 (75.0%)	1 (14.3%)
HER2-targeted therapy	2 (8.7%)	1 (6.2%)	1 (14.3%)
Others	2 (8.7%)	0	2 (28.6%)

**Fig. 1:**
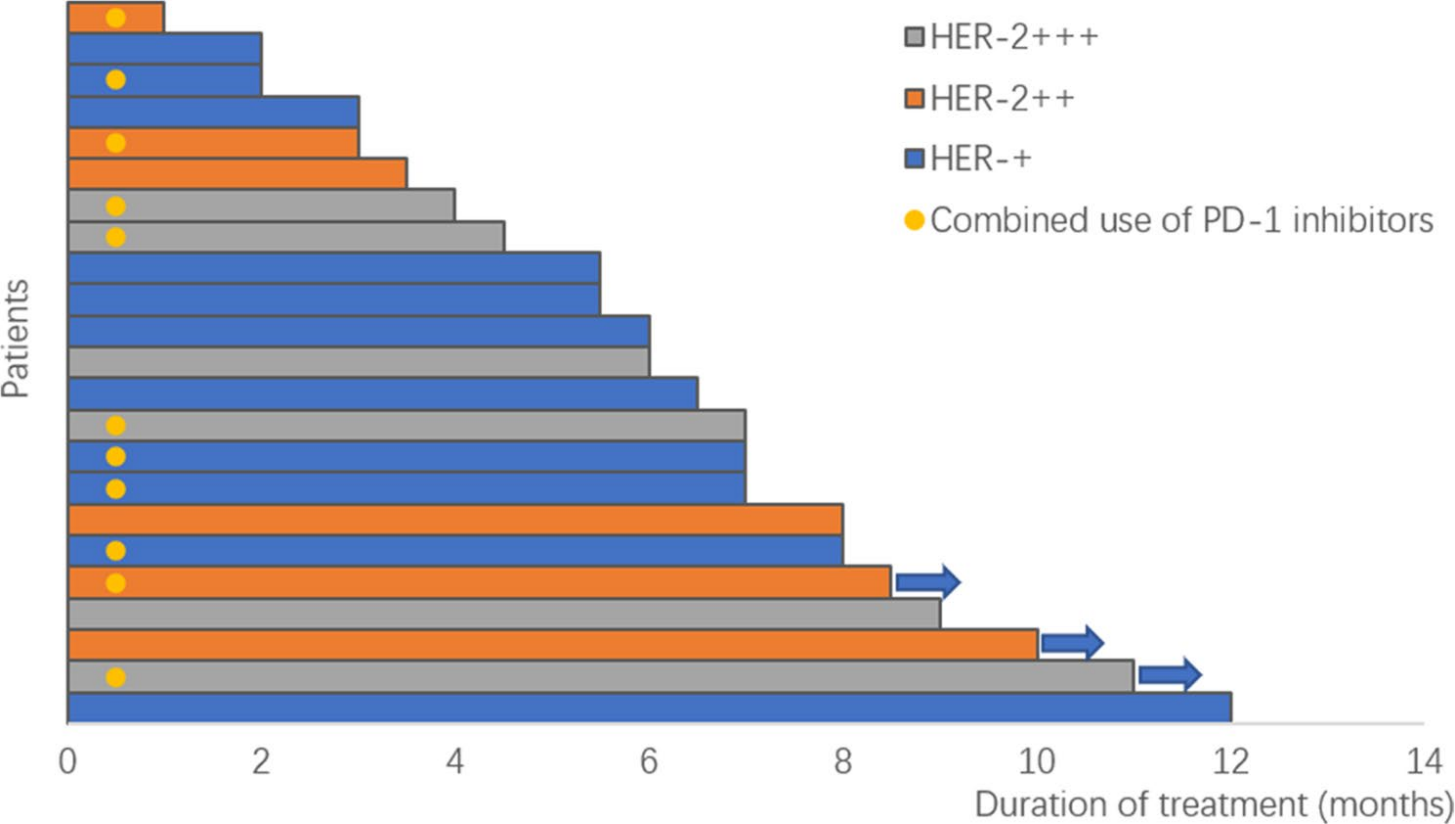
All patients in the HER2 expression cohort received treatment at a dose of 2.4 mg/kg. The dots represent patients who were treated in combination with a PD-1 inhibitor, while the arrows indicate patients who were still undergoing treatment as of the cutoff date

**Fig. 2:**
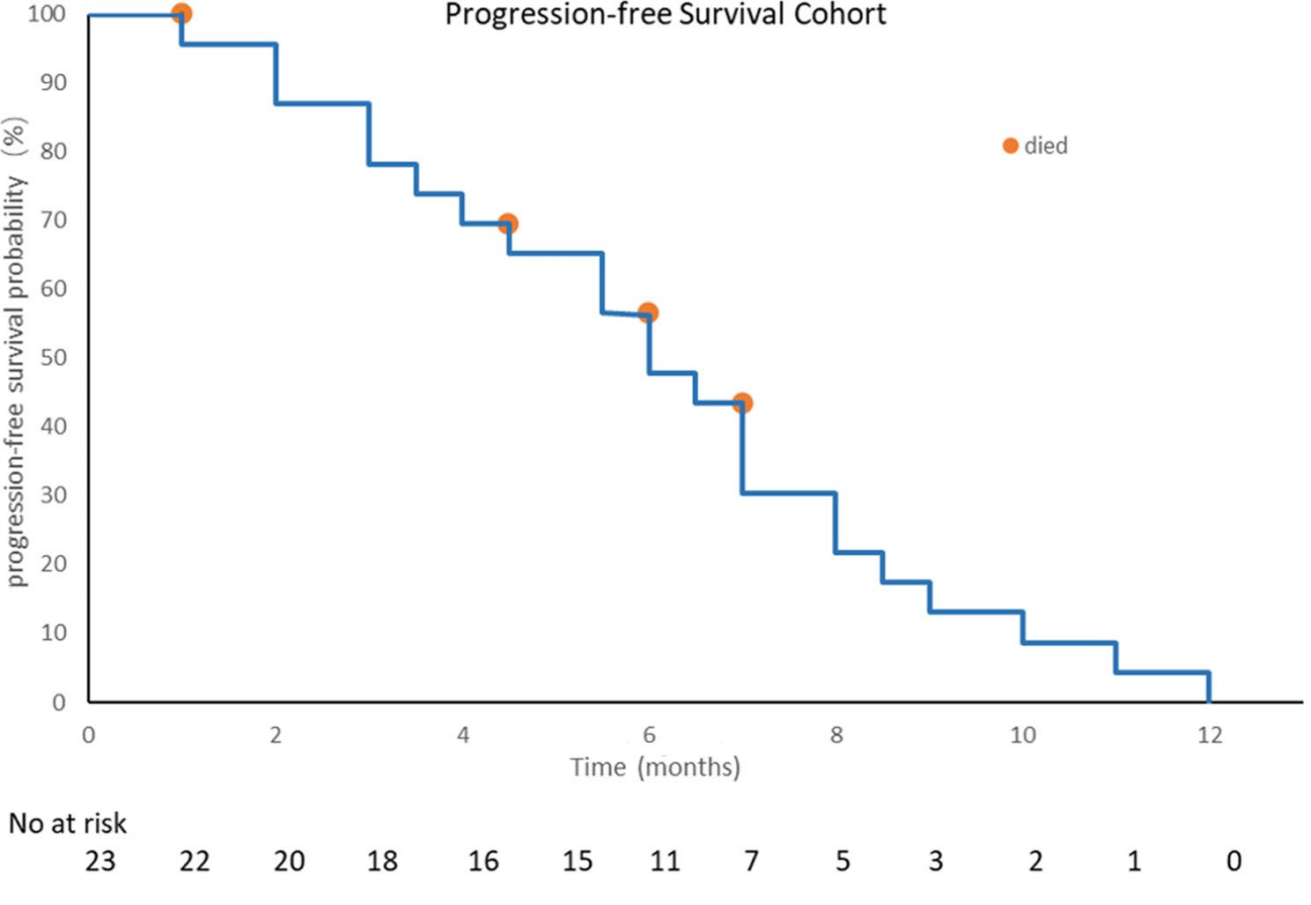
Kaplan-Meier estimates of progression-free survival (PFS).

**Fig. 3:**
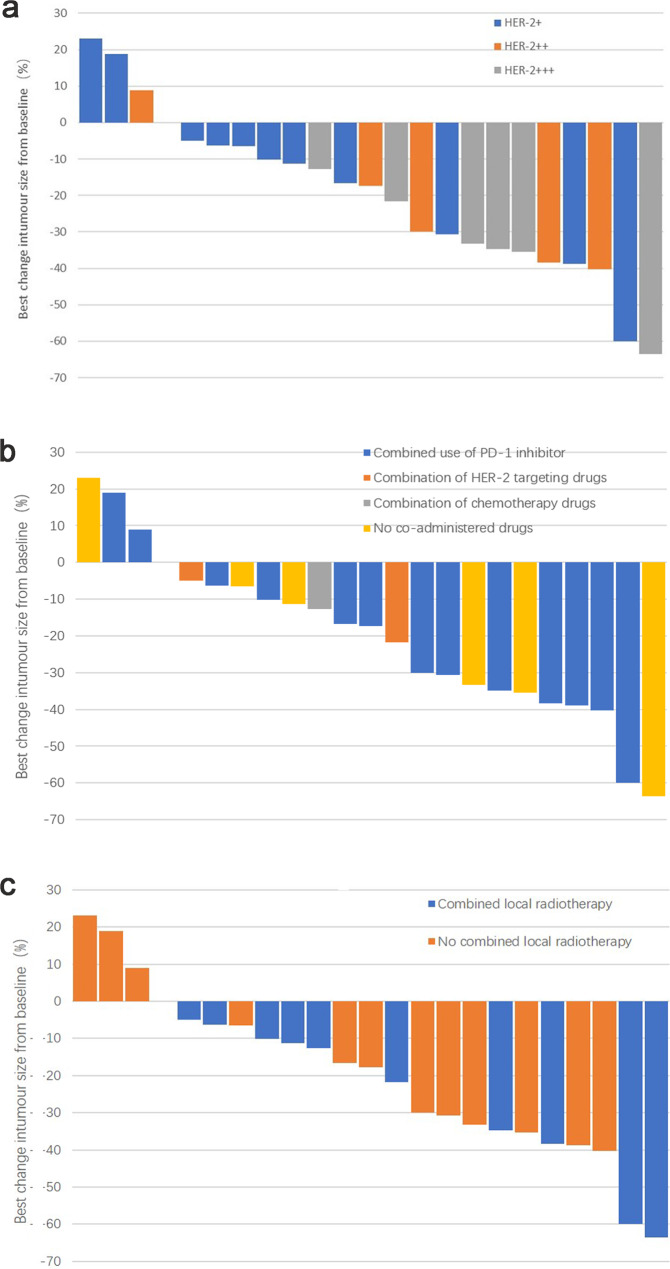
The best percentage change in tumor size relative to the baseline in target lesions of evaluable patients. The best percentage change in tumor size relative to the baseline in target lesions of evaluable patients, which is categorized by (**a**) HER2 expression, (**b**) combination with other drugs, or (**c**) combination with radiation therapy

As of December 2022, with a median follow-up of 15 months, according to the RECIST 1.1 evaluation criteria, among the patients, no one achieved CR, 10 (43.5%) had a PR, 12 (52.2%) had SD, and 1 (4.3%) showed progressive disease (PD). The ORR was 43.5% (95% CI, 21.6-65.4%), and the DCR was 95.6% (95% CI, 87.4-98.6%; Fig. 1), with a median PFS of 6.0 months (95% CI, 4.8–7.4; Fig. 2). Among the 23 patients, 19 (82.6%) exhibited a reduction in target lesions compared to baseline (Fig. 3).

In the breast cancer subgroup, 55.6% (5/9) of patients achieved confirmed objective remission, with a median PFS of 6 months (95% CI, 3.7–8.5). Among breast cancer patients, those with HER2 IHC 3 + demonstrated the most significant anti-tumor response. For the gastric cancer subgroup, the ORR stood at 57.1%, and the median PFS was 7 months (95% CI, 4.4–10.4).

In the subgroup with HER-2 low expression (ICH1+/IHC2+), the ORR was 37.5%, the median PFS spanned 5.75 months, and the DCR reached 93.7%. Conversely, in the subgroup displaying HER-2 overexpression (IHC3+), the ORR was 57.1%, the median PFS extended to 7 months, and the DCR was a comprehensive 100%.

As of the data cut-off date, 3 patients continue to receive treatment, and 19 patients are under safety survival follow-up.

During the treatment process, all patients experienced at least one treatment-related adverse event (TRAE). Bone marrow suppression emerged as the most common of these adverse events, with most cases classified as grade 1/2, including anemia (60.8%), leukopenia (56.2%), and neutropenia (43.5%). Moreover, 52.2% of patients experienced a grade 1 increase in transaminase levels. The most common severe adverse reactions, classified as grade 3 or above, were anemia (21.7%) and neutropenia (17.4%). Both these conditions showed improvement following symptomatic treatment (Table 2).

**Table 2:** Treatment-Related Adverse Events in the Study Population

	All patients (N = 23)	
	All grades	Grade ≥ 3
Leukopenia	13 (56.5%)	4 (21.7%)
Hypoesthesia	12 (52.2%)	1 (4.4%)
Neutrophil count decreased	10 (43.5%)	3 (13.0%)
Fatigue	10 (43.5%)	1 (4.4%)
Aspartate aminotransferase level increased	12 (52.2%)	1 (4.4%)
Alanine aminotransferase level increased	11 (47.8%)	0
Decreased appetite	8 (34.8%)	0
Anemia	14 (60.8%)	4 (21.7%)
Platelet count decreased	7 (30.4%)	1 (4.4%)
Nausea	8 (34.8%)	1 (4.4%)
Alopecia	10 (43.5%)	0
Weight decreased	7 (30.4%)	0
Blood triglycerides increased	7 (30.4%)	1 (4.4%)
Blood bilirubin increased	3 (13.0%)	0
Constipation	3 (13.0%)	0
Diarrhea	3 (13.0%)	0
Vomiting	3 (13.0%)	0
g-Glutamyl transferase increased	3 (13.0%)	1 (4.4%)
Pruritus	1 (4.4%)	0
Peripheral neuropathy	2 (8.7%)	0
Rash	2 (8.7%)	0
Fever	2 (8.7%)	0
Abdominal pain	2 (8.7%)	0
Arthralgia	1 (4.4%)	0
Myalgia	1 (4.4%)	0

Note: Data are presented as n (%). Adverse Events of Grade 4 or 5 were not observed

Although bone marrow suppression is a principal toxic effect of RC48, its incidence aligns with that of other chemotherapy protocols, and there were no severe adverse reactions observed. All conditions improved following suitable treatment. In this study, four cases (21.7%) presented with grade 3 anemia, two of which were patients with advanced gastric cancer with a history of long-term hematochezia. Thus, the etiology of anemia cannot be excluded as being due to the primary lesion.No reports of treatment-related interstitial lung injury were recorded in this study, suggesting good efficacy and safety.

## Discussion

HER2 is a well-established therapeutic target, with numerous targeted drugs approved for HER2-positive (IHC 3 + or 2 + and FISH +) breast cancer and gastric cancer. Overexpression and/or mutations of HER2 are observed in a variety of solid tumors, including approximately 15–20% of breast cancers [16], around 20% of gastric cancers [17], about 12% of bladder cancers [18], and roughly 25–30% of endometrial cancers [19]. For late-stage breast and gastric cancers positive for HER2, chemotherapy in combination with trastuzumab is a standard first-line treatment [20]. According to the results of the phase III ToGA trial, trastuzumab is the sole approved first-line therapy for late-stage gastric cancer overexpressing HER2 [21, 22]. Other molecular drugs targeting HER2, such as lapatinib, have shown limited effectiveness in previously treated late-stage GC that is HER2 positive [23]. Currently, third-line treatment options for late-stage GC patients encompass chemotherapy (irinotecan, paclitaxel, and TAS-102), immunotherapy (pembrolizumab), and targeted therapy (apatinib). However, the ORR is generally low (2.84-11.6%), with median PFS ranging from approximately 1.6 to 5.8 months [24–28]. Over the past several decades, there has been no opportunity for targeted therapy for late-stage malignant tumors with medium to low expression of HER2, with chemotherapy being the primary treatment method [29]. The median PFS is 1.9-5.3 m and OS for patients undergoing chemotherapy alone is less than a year. RC48-C008 is a single-arm, open-label, multi-center phase II clinical trial of trastuzumab deruxtecan in patients with gastric/gastroesophageal junction adenocarcinoma, including those who have previously undergone ≥ 2 lines of chemotherapy for HER2 overexpression (IHC 2 + or IHC 3+). The ORR reached 24.8%, the DCR was 42.4%, the median PFS was 4.1 months, and the median OS was 7.9 months [10].

In this study, we analyzed the effectiveness and safety of RC48 as a second-line or beyond treatment for patients with HER2-expressing advanced or metastatic solid tumors in real-world settings. The results have been promising. Among the 23 patients, 10 (43.5%, 95% CI, 21.6-65.4%) achieved a PR, and approximately 90.7% of patients had a positive therapeutic response, obtaining satisfactory disease control. Both the ORR and the DCR significantly surpassed those of other approved second- or third-line treatments (Supplemented Table 1). Consequently, RC48 may provide a novel treatment avenue for patients with HER2-expressing tumors after the progression of chemotherapy and immunotherapy.

Further stratified analysis revealed that among eight patients treated with second-line therapy, the ORR and DCR were 46.7% and 100.0%, respectively, with a PFS of 6.25 months. Among 15 patients treated with third-line or beyond who received RC48 treatment, ORR, and DCR were 46.7% and 93.3% respectively. Of these, seven patients who progressed after anti-HER2 monoclonal antibody targeted therapy achieved PR in three cases (42.8%) after RC48-ADC treatment. Therefore, patients with HER2 overexpression may still benefit from RC48 after progression following anti-HER2 monoclonal antibody treatment. The underlying reasons for this are firstly, the humanized anti-HER2 antibody hertuzumab in RC48 is linked to MMAE through a cleavable linker. In addition to inhibiting the HER2 receptor signaling pathway, RC48 also exerts anti-tumor effects through MMAE-induced cytotoxicity. Preclinical studies have indicated antibody-dependent cell-mediated cytotoxicity (ADCC) activity of RC48 [5, 30, 31]. Secondly, the antibody in RC48 has a higher affinity for HER2 [31], effectively targeting HER2-expressing tumor cells. Finally, the MMAE released by enzymatic cleavage has high membrane permeability, can penetrate adjacent cells to produce a bystander effect, and has therapeutic effects on tumor cells with low or no HER2 expression [32, 33]. Furthermore, in the RC48-C011 and RC48-C008 clinical trials [32], similar conclusions were drawn: for HER2 negative (IHC 0 or 1+) advanced urothelial cancer (UC) patients who had previously undergone ≥ 1 systemic treatment, ORR was 26.3%. For HER2 positive late-stage gastric cancer patients who had previously received ≥ 2 systemic treatments, ORR was 24.8% [10].

In this study, we also observed another encouraging phenomenon: RC48-ADC demonstrated rapid clinical responses, with a median response time of 42 days. This swift clinical response effectively reduces tumor burden, alleviates patient symptoms, improves quality of life, and provides an effective time window and good physical reserves for tolerating comprehensive anti-tumor treatments.

The treatment of tumors emphasizes comprehensive therapy, especially for patients with advanced or metastatic tumors. In this study, the majority of patients adopted a combination therapy approach, including local radiotherapy, PD-1 inhibitors, and anti-angiogenic therapy, among others. Among the ten patients who received RC48 combined with radiation therapy, all observed tumor shrinkage, and RC48 combined with local radiotherapy had better disease control, reaching 100%. However, for the 13 patients who did not receive combined radiation therapy, only 69.2% observed tumor shrinkage.

In preclinical investigations, trastuzumab has been shown to provoke HER2-specific T-cell responses [34] and escalate the expression of Programmed Death-Ligand 1 (PD-1) and PD-L1 [35, 36]. Concurrent administration of a PD-1 inhibitor and trastuzumab enhances the HER2-specific T-cell responses [25, 34], and facilitates the development of immunological memory for effective tumor eradication, thereby safeguarding against tumor relapse [37]. Additionally, anti-HER2 targeted ADC have exhibited selective radiosensitizing effects [38]. Based on these insights, a subset of patients were subjected to a combination therapy of RC-48 and a PD-1 inhibitor. Among these 13 patients, the ORR and the DCR were observed to be 53.8% and 84.6%, respectively, thereby significantly outperforming the results of the Phase Ib trial of T-DXd and Nivolumab, presented at the 2022 ASCO-GU annual meeting (ORR: 36.7% [11/30], median mPFS: 6.9 months) [39]. In the subset of 10 patients undergoing combination therapy with RC48 and radiation, tumor reduction was observed across all cases. However, in the 13 patients who did not receive radiation therapy, tumor reduction was seen only in 69.2% of the cases.

In the subgroup of patients with breast cancer, 55.6% (5/9) achieved a confirmed objective remission, with a PFS of 6 months (95% CI, 3.7–8.5). Among these patients, those with HER2 IHC 3 + displayed the most significant anti-tumor response. Beyond breast cancer, our findings also highlight the primary efficacy of RC48 in other solid tumors expressing HER2. Notably, for gastric cancer characterized by low HER-2 expression, we observed an ORR of 57.1% (4/7) and a median PFS of 7 months (95% CI, 4.4–10.4). Gastric cancer patients exhibited a better PFS compared to those with breast cancer. This disparity might be attributed to the fact that the average number of prior systemic treatment lines in the gastric cancer subgroup was fewer than that in the breast cancer subgroup (1.6 vs. 3.8). In the gastric cancer cohort, 71.4% (5/7) of patients received combination therapy with the PD-1 inhibitor, Cedilimumab, whereas only 22.2% (2/9) of the breast cancer cohort were treated with the same. This supports the therapeutic advantages of combining RC48 with immunotherapy. The sole colorectal cancer patient reported exhibiting stable disease. Given the limited sample size of colorectal cancer patients in our study, it is not feasible to draw definitive conclusions regarding efficacy. A more comprehensive investigation is warranted, focusing on colorectal cancer patients exhibiting HER2 overexpression.

Compared with second-line chemotherapy regimens for advanced or metastatic solid tumors, with an mPFS of approximately 1.9–5.3 months [26–28], trastuzumab deruxtecan demonstrated superior efficacy both in combination with a PD-1 inhibitor and as a monotherapy, with manageable safety. Furthermore, RC48 and PD-1 inhibitors show a beneficial synergistic effect, significantly superior to the efficacy of using RC48 or PD-1 inhibitors alone [40]. Among the 13 patients combined with PD-1 inhibitors in the study, the ORR was 53.8%, and the DCR was 84.6%. This is notably superior to the phase IB study combining T-DXd and Nivolumab presented at the 2022 ASCO-GU annual meeting [41] (ORR of 36.7% (11/30) and mPFS of 6.9 months). RC48, whether combined with PD-1 inhibitors or used alone, demonstrates excellent therapeutic efficacy, with manageable safety. Firstly, the data on RC48 combined with PD-1 indicated that patients with HER2-expressing advanced solid tumors can benefit regardless of the line of treatment, HER2, and PD-L1 expression status. This study also confirms that RC48 combined with immunotherapy could potentially become a leader in the field of combined treatment for HER2-expressing advanced solid tumors.

In the present study, repeat biopsies were conducted during treatment to confirm the HER2 status in tumor tissues for nine patients. Post-treatment, five patients (55.6%) exhibited a decline in HER2 expression levels (HER2 IHC1 + changed to HER2(-) in three patients, HER2 IHC2 + changed to HER2(-) in one patient, and HER2 IHC3 + changed to HER2 IHC2 + in one patient). These observations indicate temporal heterogeneity in tumor HER2 expression and a loss of HER2 protein following treatment. Research by Pietrantonio et al. [42] demonstrated that approximately 32% of HER2-positive gastric cancer patients exhibited HER2 loss following initial treatment with trastuzumab. Yoshimoto et al. [43] suggested that HER2 loss might occur due to alterations in HER2 during tumor progression. This implies that the expression status of HER2 could be influenced by chemotherapy and/or anti-HER2 treatment, leading to changes in pre- and post-treatment HER2 status. Therefore, dynamic assessment of HER2 expression changes is required in the holistic management of patients, to determine the applicability of HER2-targeted treatment in the event of disease progression.

RC48-ADC demonstrated consistent clinical activity across all HER2-expressing subgroups. Among the patients with low/medium HER2 expression (ICH 1+/2+), representing 69.6% (16 cases) of the group, the ORR was 37.5%, with a median PFS of 5.75 months and a DCR of 93.7%. For the seven patients (30.4%) with high HER2 expression (ICH 3+), the ORR was 57.1%, with a median PFS of 7 months and a DCR of 100%. Once RC48 targets HER2-expressing tumor cells, the enzymatically-released MMAE with high membrane permeability can penetrate neighboring cells to produce a bystander effect, thereby killing tumor cells that do not express HER2 [31]. For patients with low HER2 expression who, under the previous one-size-fits-all treatment approach (HER2 positive being ICH 3 + or ICH 2+, FSIH (+)), had no opportunity for targeted therapy, they can now benefit from second- or third-line RC48-ADC treatment following progression from chemotherapy and/or immunotherapy. The efficacy in patients with high expression was slightly better than in those with low to medium expression. RC48 showcases superiority in treating both high and low HER2-expressing advanced solid tumors, exceeding the therapeutic efficacy of existing treatments for advanced solid tumors [27, 28, 44, 45], thus filling a global unmet need. Furthermore, the data indicates that RC48-ADC is effective in HER2 low-expressing populations, proving its characteristic of high HER2 affinity and potent bystander effect. This implies that RC48-ADC might gain an advantage in various solid tumors with low HER2 expression.

## Conclusion

In our comprehensive real-world analysis, we determined that RC48, irrespective of HER2 expression levels, offers a notable advantage in efficacy and safety profiles when compared to traditional treatments. Specifically, when used in tandem with PD-1 inhibitors or as a standalone therapy in second-line or subsequent treatments, RC48 showcased promising results. Representing a new paradigm in ADC therapies, RC48 paves the way for innovative treatments targeting HER2-expressing advanced or metastatic tumors. However, it’s important to note the limitations of our study. The relatively small sample size may impact the generalizability of our findings, and we recommend larger-scale studies to validate and further elucidate the potential of RC48. Despite these limitations, our study underscores the necessity for further investigative studies and highlights the potential of RC48 in transforming the therapeutic landscape.

### Electronic supplementary material

Below is the link to the electronic supplementary material.

Supplementary Material 1

## Data Availability

The datasets generated during and/or analyzed during the current study are available from the corresponding author on reasonable request.
