# Supplementary material for: RC48-ADC treatment for patients with HER2-expressing locally advanced or metastatic solid tumors: a real-world study
Source: BMC Cancer. 2023 Nov 9;23:1083. doi: 10.1186/s12885-023-11593-9 (PMC10636982; doi:10.1186/s12885-023-11593-9)
Supplement: Supplementary file 1 — Supplementary Material 1 [file 12885_2023_11593_MOESM1_ESM.docx]

**Supplemented Table 1**. Clinical study of gastric cancer, breast cancer and urothelial carcinoma.

| Indication | Clinical stage  (Trial) | Clinical Protocol | Number of participants (n) | mPFS (months) | mOS (months) | ORR | DCR | Registration number |
| --- | --- | --- | --- | --- | --- | --- | --- | --- |
| Gastroesophageal cancer / second-line^[1]^ | Phase III  (ABSOLUTE) | Nab-paclitaxel *vs.* weekly nab-paclitaxel *vs.* solvent-based paclitaxel | 741 | 3.8 *vs.* 5.3 *vs.* 3.8 | 10.3 *vs.* 11.1 *vs.* 10.9 | 25% *vs.* 33% *vs.* 24% | 66% *vs.* 74% *vs.* 70% | NCT04294784 |
| Gastroesophageal cancer / second-line^[2]^ | Phase II | Epirubicin+Oxaliplatin+  Capecitabine *vs.* Docetaxel+Oxaliplatin *vs.* Oxaliplatin | 55 | 5.4 *vs.* 5.6 *vs.* 3.1 | 8.1 *vs.* 9.5 *vs.* 3.6 | 29% *vs.* 47% *vs.* 11% | 59% *vs.* 58% *vs.* 44% | NCT01710592 |
| Gastroesophageal cancer / second-line^[3]^ | Phase I/II  (POLARIS-02) | Toripalimab | 58 | 1.9 | 4.8 | 12.1% | 39.7% | NCT02915432 |
| Gastroesophageal cancer / second-line^[4]^ | Phase III  (RAINBOW) | Ramucirumab plus paclitaxel *vs.* Placebo plus paclitaxel | 665 | 4.2 *vs.* 2.9 | 8.5 *vs.* 5.9 | 28% *vs.* 17% | 80% *vs.* 63% | NCT01170663 |
| Gastroesophageal cancer /third-line^[5]^ | Phase III | Apatinib *vs.* Placebo | 224 | 2.6 *vs.* 1.8 | 6.5 *vs.* 4.7 | 2.84% *vs.* 0 | 42.05% *vs.* 8.79% | NCT01512745 |
| Gastroesophageal cancer /third-line^[6]^ | Phase III  (ATTRACTION-2) | Nivolumab *vs.* Placebo | 493 | 1.61 *vs.* 1.45 | 5.26 *vs.* 4.14 | 11% *vs.* 0 | 40% *vs.* 25% | NCT02267343 |
| Breast cancer/ third-line^[7]^ | Phase III  (340 study) | Eribulin *vs.* Vinorelbine | 530 | 2.8 *vs.* 2.8 | 13.4 *vs.* 12.5 | 30.7% *vs.* 16.9% | 49.2% *vs.* 33.1% | NCT02225470 |
| Breast cancer/ third-line^[8]^ | Phase III  (ASCENT) | Sacituzumab govitecan | 529 | 4.8 *vs.* 1.7 | 11.8 *vs.* 6.9 | 31% *vs.* 4% | 67% *vs.* 31% | NCT02574455 |
| Breast cancer/ second /third-line^[9]^ | Phase III  (KEYNOTE-119) | Pembrolizumab *vs.* capecitabine/eribulin/gemcitabine | 622 | / | 9.9 *vs.* 10.8 | 9.6% *vs.* 10.6% | 12.2% *vs.* 18.7% | [NCT02555657](http://clinicaltrials.gov/show/NCT02555657) |
| Urothelial carcinoma/second-line^[10]^ | Phase III  (KEYNOTE-045) | Pembrolizumab *vs.* paclitaxel/docetaxel/or vinflunine | 542 | 2.1 *vs.* 3.3 | 10.3 *vs.* 7.4 | 21.1% *vs.* 11.6% | 38.5% *vs.* 44.9% | [NCT02256436](http://clinicaltrials.gov/show/NCT02256436) |
| Urothelial carcinoma/second-line^[11]^ | Phase II  (Checkmate275) | Nivolumab | 270 | 1.9 | 8.6 | 20.7% | 41.4% | NCT03219775 |
| Urothelial carcinoma/second-line^[12]^ | Phase III  (RANGE) | Ramucirumab+docetaxel *vs.* Placebo+docetaxel | 530 | 4.1 *vs.* 2.8 | 9.4 *vs.* 7.9 | 25.9% *vs.* 13.9% | 65.4% *vs.* 55.1% | NCT02426125 |
| Urothelial carcinoma/second-line^[13]^ | Phase II  (BLC2001) | Erdafitinib | 99 | 5.5 | 13.8 | 40% | 79% | NCT02365597 |
| Urothelial carcinoma/≥second-line^[14]^ | Phase II  (RC48-C005) | Disitamab Vedotin | 43 | 6.9 | 13.9 | 51.2% | 90.7% | NCT03507166 |
| Urothelial carcinoma/ third-line^[15]^ | Phase II  (TROPHY-U-01) | Sacituzumab govitecan | 113 | 4.95 | 9.95 | 27% | 61% | NCT03547973 |

**References:**

1. Shitara K, Takashima A, Fujitani K, Koeda K, Hara H, Nakayama N, Hironaka S, Nishikawa K, Makari Y, Amagai K et al: Nab-paclitaxel versus solvent-based paclitaxel in patients with previously treated advanced gastric cancer (ABSOLUTE): an open-label, randomised, non-inferiority, phase 3 trial. The lancet Gastroenterology & hepatology 2017, 2(4):277-287.

2. Hall PS, Lord SR, Collinson M, Marshall H, Jones M, Lowe C, Howard H, Swinson D, Velikova G, Anthoney A et al: A randomised phase II trial and feasibility study of palliative chemotherapy in frail or elderly patients with advanced gastroesophageal cancer (321GO). Br J Cancer 2017, 116(4):472-478.

3. Wang F, Wei XL, Wang FH, Xu N, Shen L, Dai GH, Yuan XL, Chen Y, Yang SJ, Shi JH et al: Safety, efficacy and tumor mutational burden as a biomarker of overall survival benefit in chemo-refractory gastric cancer treated with toripalimab, a PD-1 antibody in phase Ib/II clinical trial NCT02915432. Annals of oncology : official journal of the European Society for Medical Oncology 2019, 30(9):1479-1486.

4. Wilke H, Muro K, Van Cutsem E, Oh SC, Bodoky G, Shimada Y, Hironaka S, Sugimoto N, Lipatov O, Kim TY et al: Ramucirumab plus paclitaxel versus placebo plus paclitaxel in patients with previously treated advanced gastric or gastro-oesophageal junction adenocarcinoma (RAINBOW): a double-blind, randomised phase 3 trial. The Lancet Oncology 2014, 15(11):1224-1235.

5. Li J, Qin S, Xu J, Xiong J, Wu C, Bai Y, Liu W, Tong J, Liu Y, Xu R et al: Randomized, Double-Blind, Placebo-Controlled Phase III Trial of Apatinib in Patients With Chemotherapy-Refractory Advanced or Metastatic Adenocarcinoma of the Stomach or Gastroesophageal Junction. Journal of clinical oncology : official journal of the American Society of Clinical Oncology 2016, 34(13):1448-1454.

6. Kang YK, Boku N, Satoh T, Ryu MH, Chao Y, Kato K, Chung HC, Chen JS, Muro K, Kang WK et al: Nivolumab in patients with advanced gastric or gastro-oesophageal junction cancer refractory to, or intolerant of, at least two previous chemotherapy regimens (ONO-4538-12, ATTRACTION-2): a randomised, double-blind, placebo-controlled, phase 3 trial. Lancet (London, England) 2017, 390(10111):2461-2471.

7. Yuan P, Hu X, Sun T, Li W, Zhang Q, Cui S, Cheng Y, Ouyang Q, Wang X, Chen Z et al: Eribulin mesilate versus vinorelbine in women with locally recurrent or metastatic breast cancer: A randomised clinical trial. European journal of cancer (Oxford, England : 1990) 2019, 112:57-65.

8. Bardia A, Hurvitz SA, Tolaney SM, Loirat D, Punie K, Oliveira M, Brufsky A, Sardesai SD, Kalinsky K, Zelnak AB et al: Sacituzumab Govitecan in Metastatic Triple-Negative Breast Cancer. The New England journal of medicine 2021, 384(16):1529-1541.

9. Winer EP, Lipatov O, Im SA, Goncalves A, Muñoz-Couselo E, Lee KS, Schmid P, Tamura K, Testa L, Witzel I et al: Pembrolizumab versus investigator-choice chemotherapy for metastatic triple-negative breast cancer (KEYNOTE-119): a randomised, open-label, phase 3 trial. The Lancet Oncology 2021, 22(4):499-511.

10. Bellmunt J, Bajorin DF: Pembrolizumab for Advanced Urothelial Carcinoma. The New England journal of medicine 2017, 376(23):2304.

11. Grimm MO, Grün CB, Niegisch G, Pichler M, Roghmann F, Schmitz-Dräger B, Baretton G, Schmitz M, Bolenz C, Foller S et al: Tailored immunotherapy approach with nivolumab with or without ipilimumab in patients with advanced transitional cell carcinoma after platinum-based chemotherapy (TITAN-TCC): a multicentre, single-arm, phase 2 trial. The Lancet Oncology 2023, 24(4):347-359.

12. van der Heijden MS, Powles T, Petrylak D, de Wit R, Necchi A, Sternberg CN, Matsubara N, Nishiyama H, Castellano D, Hussain SA et al: Predictive biomarkers for survival benefit with ramucirumab in urothelial cancer in the RANGE trial. Nature communications 2022, 13(1):1878.

13. Loriot Y, Necchi A, Park SH, Garcia-Donas J, Huddart R, Burgess E, Fleming M, Rezazadeh A, Mellado B, Varlamov S et al: Erdafitinib in Locally Advanced or Metastatic Urothelial Carcinoma. The New England journal of medicine 2019, 381(4):338-348.

14. Kannan A, Wells RB, Sivakumar S, Komatsu S, Singh KP, Samten B, Philley JV, Sauter ER, Ikebe M, Idell S et al: Mitochondrial Reprogramming Regulates Breast Cancer Progression. Clinical cancer research : an official journal of the American Association for Cancer Research 2016, 22(13):3348-3360.

15. Tagawa ST, Petrylak DP, Grivas P, Agarwal N, Sternberg CN, Siemon-Hryczyk P, Goswam T, Loriot Y: TROPHY-u-01: A phase II open-label study of sacituzumab govitecan (IMMU-132) in patients with advanced urothelial cancer after progression on platinum-based chemotherapy and/or anti-PD-1/PD-L1 checkpoint inhibitor therapy. 2019, 37(7_suppl):TPS495-TPS495.
